# Particle-size dependent structural transformation of skyrmion lattice

**DOI:** 10.1038/s41467-020-19480-8

**Published:** 2020-11-11

**Authors:** R. Takagi, Y. Yamasaki, T. Yokouchi, V. Ukleev, Y. Yokoyama, H. Nakao, T. Arima, Y. Tokura, S. Seki

**Affiliations:** 1 RIKEN Center for Emergent Matter Science (CEMS), Wako, 351-0198 Japan; 2 Department of Applied Physics, University of Tokyo, Tokyo, 113-8656 Japan; 3 Institute of Engineering Innovation, University of Tokyo, Tokyo, 113-0032 Japan; 4 Research and Services Division of Materials Data and Integrated System (MaDIS), National Institute for Materials Science (NIMS), Tsukuba, 305-0047 Japan; 5 PRESTO, Japan Science and Technology Agency (JST), Kawaguchi, 332-0012 Japan; 6 Photon Factory, Institute of Materials Structure Science, High Energy Accelerator Research Organization, Tsukuba, 305-0801 Japan; 7 Laboratory for Neutron Scattering and Imaging (LNS), Paul Scherrer Institute (PSI), CH-5232 Villigen, Switzerland; 8 Japan Synchrotron Radiation Research Institute (JASRI/SPring-8), Sayo, 679-5198 Japan; 9 Department of Advanced Materials Science, University of Tokyo, Kashiwa, 277-8561 Japan; 10 Tokyo College, University of Tokyo, Tokyo, 113-8656 Japan

**Keywords:** Magnetic properties and materials, Topological matter

## Abstract

Magnetic skyrmion is a topologically protected particle-like object in magnetic materials, appearing as a nanometric swirling spin texture. The size and shape of skyrmion particles can be flexibly controlled by external stimuli, which suggests unique features of their crystallization and lattice transformation process. Here, we investigated the detailed mechanism of structural transition of skyrmion lattice (SkL) in a prototype chiral cubic magnet Cu_2_OSeO_3_, by combining resonant soft X-ray scattering (RSXS) experiment and micromagnetic simulation. This compound is found to undergo a triangular-to-square lattice transformation of metastable skyrmions by sweeping magnetic field (*B*). Our simulation suggests that the symmetry change of metastable SkL is mainly triggered by the *B-*induced modification of skyrmion core diameter and associated energy cost at the skyrmion-skyrmion interface region. Such internal deformation of skyrmion particle has further been confirmed by probing the higher harmonics in the RSXS pattern. These results demonstrate that the size/shape degree of freedom of skyrmion particle is an important factor to determine their stable lattice form, revealing the exotic manner of phase transition process for topological soliton ensembles in the non-equilibrium condition.

## Abstract (web summary)

Skyrmions are topological spin textures and are of great interest due to their impressive stability. Here, by sweeping an applied magnetic field, the authors observe a change in the skyrmion lattice structure, shedding light on the relation between skyrmion size and stability.

## Introduction

**Fig. 1:**
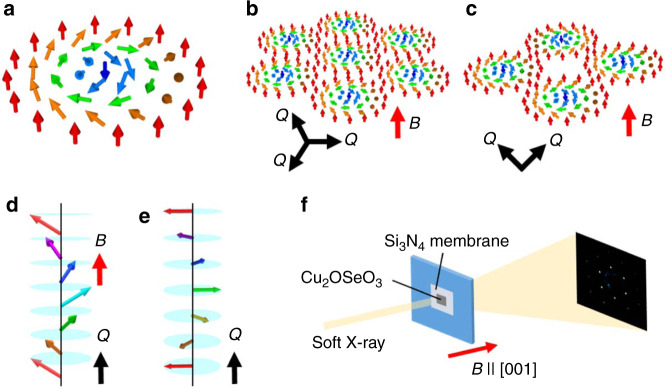
Small-angle resonant soft X-ray scattering (RSXS) experiments for the detection of various spin textures. **a**–**e** Schematic illustrations of an isolated skyrmion (**a**), triangular SkL (**b**), square SkL (**c**), conical (**d**), and helical (**e**) spin textures. The directions of magnetic modulation vector **Q** and magnetic field *B* are also indicated. **f** Experimental setup for the small-angle RSXS measurements (see “Methods”).

Recently, the concept of topology attracts attention as a source of rich emergent phenomena in condensed matters. In the systems with tensor fields, singular defects called topological solitons often appear, which cannot be erased by the continuous deformation and therefore behave as stable objects^1^. One typical example is a skyrmion in magnetic materials^2–6^, which appears as a nanometric swirling spin texture with particle-like character as shown in Fig. 1a. Skyrmion spin texture is generally characterized by non-zero integer skyrmion number *N*_sk_ described by^6^

1 $$N_{{\mathrm{sk}}} = {\int} {n_{{\mathrm{sk}}}dxdy} = \frac{1}{{4\pi }}{\int} {{\mathbf{n}} \cdot \left( {\frac{{\partial {\mathbf{n}}}}{{\partial x}} \times \frac{{\partial {\mathbf{n}}}}{{\partial y}}} \right)dxdy,}$$

which represents how many times the spin directions wrap a unit sphere. Here, the integral is taken over the two-dimensional magnetic unit cell and ${\mathbf{n}}\left( {\mathbf{r}} \right) = {\mathbf{m}}\left( {\mathbf{r}} \right)/|{\mathbf{m}}\left( {\mathbf{r}} \right)|$ and *n*_sk_ (**r**) represent the unit vector pointing in the local magnetization (**m**(**r**)) direction and topological charge density, respectively. Interestingly, similar topological solitons are also known to appear in various physical context, such as skyrmions, hopfions, and heliknotons in liquid crystals^7–10^ or Abrikosov vortices in type-II superconductors^11^. These particle-like objects generally prefer to form a periodic lattice in the similar manner as atomic or molecular crystals, implying that topological solitons can be a unique building block for a rich variety of tunable ordered structures.

Experimentally, magnetic skyrmions are found in a series of noncentrosymmetric systems, such as metallic *B*20 (MnSi, FeGe, Fe_1−*x*_Co_*x*_Si, etc.)^3,4,6^, Co-Zn-Mn alloys^12^, and insulating Cu_2_OSeO_3_^13,14^. These compounds are characterized by the chiral cubic crystal structure, where Dzyaloshinskii–Moriya (DM) interaction plays a key role in the stabilization of the skyrmion spin texture. In such systems, skyrmions usually crystallize into a close-packed triangular-lattice form in the equilibrium condition (Fig. 1b). On the other hand, recent studies on MnSi^15,16^ and Co-Zn-Mn alloys^17,18^ have revealed the reversible transition between the triangular skyrmion lattice (SkL) (Fig. 1b) and square SkL (Fig. 1c) as a function of temperature (*T*) and/or external magnetic field (*B*) in the non-equilibrium condition. For such a structural transition of SkL, the possible relevance of the magnetic anisotropy has been discussed, while the detailed mechanism is yet to be clarified.

In the present study, we have investigated the microscopic origin of such a symmetry change of magnetic SkL. By performing the small-angle resonant soft X-ray scattering (RSXS) experiments for a prototype chiral-lattice insulator Cu_2_OSeO_3_, *B-*induced triangular-to-square lattice transformation of metastable skyrmions is confirmed. Our micromagnetic simulation, without including magnetic anisotropy term, reveals that the observed SkL transformation is mainly triggered by *B-*dependent change of skyrmion core diameter and associated energy cost at the skyrmion–skyrmion interface region. Such internal deformation of skyrmion particle has indeed been detected experimentally by measuring the higher harmonics in the RSXS patterns. Our results reveal the unique manner of phase transition process of SkL in the non-equilibrium condition and suggest that the size/shape degree of freedom of skyrmion particle plays an important role in the determination of their stable lattice form.

## Results

Our target material Cu_2_OSeO_3_ is an insulator characterized by a chiral cubic crystal structure with the space group *P*2_1_3^13,14^. The magnetism is governed by the Cu^2+^ ion with *S* = 1/2 and the DM interaction leads to the long-period modulation of local magnetic moment direction. To investigate the detailed spin texture, RSXS experiments have been performed in the small-angle scattering geometry for a (001)-oriented Cu_2_OSeO_3_ thin plate (Fig. 1f). Here, the directions of incident soft X-ray beam and external magnetic field are fixed perpendicular to the sample surface (i.e., parallel to the out-of-plane [001] direction). When the Fourier transform of magnetic structure contains the modulated spin component $({\hat{\mathbf{m}}}({\mathbf{Q}})\exp [i{\mathbf{Q}} \cdot {\mathbf{r}}] + c.c.)$ with ${\hat{\mathbf{m}}}({\mathbf{Q}})$ being a complex vector, the corresponding magnetic scattering intensity *I*(**Q**) can be described as $I({\mathbf{Q}}) \propto |\left( {{\mathbf{e}}_i \times {\mathbf{e}}_f} \right) \cdot {\hat{\mathbf{m}}}({\mathbf{Q}})|^2$, where e_*i*_ and e_*f*_ represent the polarization vectors of incident and scattered beams, respectively^19^. As the scattered beam is approximately parallel to the incident beam in the small-angle scattering geometry, the component of ${\hat{\mathbf{m}}}({\mathbf{Q}})$ parallel to the out-of-plane [001] direction (i.e., $\hat m_z({\mathbf{Q}})$) is mainly detected in the present measurements.

**Fig. 2:**
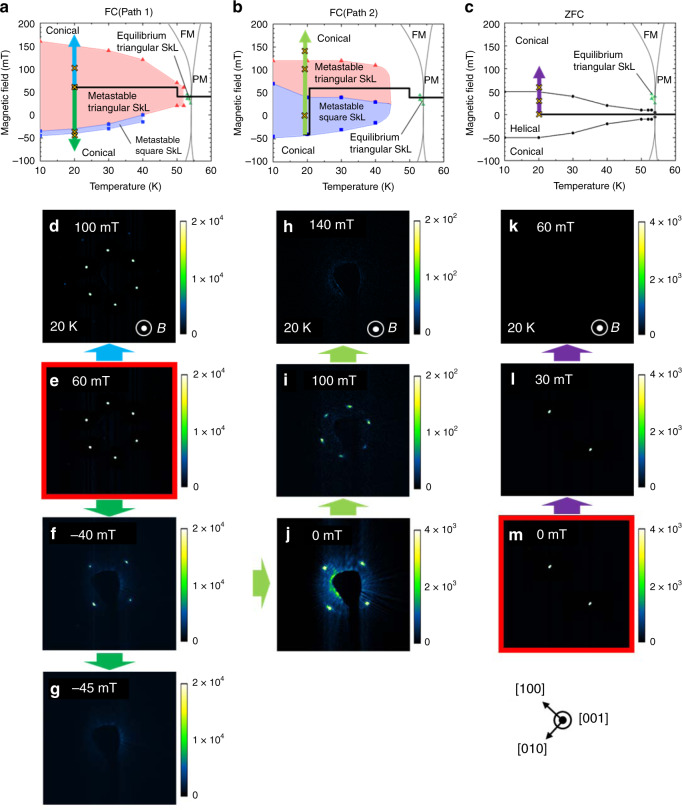
RSXS diffraction patterns obtained for various field-sweeping paths in Cu_2_OSeO_3_. **a**–**c** Magnetic field (*B*)–temperature (*T*) phase diagrams for B || [001], determined from the field-sweeping runs after the different paths of field cooling (**a**, **b**) and zero-field cooling (**c**) shown by the arrows in each figure. FM and PM represent the ferromagnetic and paramagnetic states, respectively. **d**–**m** RSXS diffraction patterns taken at 20 K with various amplitudes of magnetic field along the out-of-plane [001] direction in the field-sweeping process of Path 1 (**d**–**g**) and Path 2 (**h**–**j**) after FC and after ZFC (**k**–**m**). The color indicates the scattering intensity. Arrows between these figures represent the direction of field sweep, which correspond to the arrows in **a**–**c**.

Figure 2c indicates the *B*–*T* magnetic phase diagram for the present sample in the equilibrium condition, determined by the RSXS measurement in the field-sweeping process after a zero-field cooling (ZFC) (see Supplementary Note I for the detail). Cu_2_OSeO_3_ hosts helical magnetic order (Fig. 1e) at *B* = 0, where the neighboring spins rotate within a plane normal to the magnetic modulation vector **Q** || < 100 > . The application of a magnetic field along the out-of-plane [001] direction induces the transition into the conical magnetic phase (Fig. 1d), in which the **Q***-*vector is aligned parallel to the magnetic field (i.e., **Q** *||* **B**). The equilibrium triangular SkL phase (Fig. 1b) appears in the narrow *B*–*T* region just below the magnetic ordering temperature *T*_c_, where the spin texture can be approximately described as ${\mathbf{m}}({\mathbf{r}}) = \left( {0,0,m_0^z} \right) + \mathop {\sum}\nolimits_{\upsilon = 1,2,3} {({\hat{\mathbf{m}}}({\mathbf{Q}}_{\upsilon})\exp \left[ {i{\mathbf{Q}}_\upsilon \cdot {\mathbf{r}}} \right] + c.c)}$, with $m_0^z$ being the *B*-induced out-of-plane uniform magnetization component. In this situation, the three *Q*-vectors lie perpendicular to the magnetic field (i.e., **Q** ⊥ **B**). The observed *B*–*T* phase diagram in Fig. 2c is consistent with the previous reports^13,14^. It is noteworthy that the low-temperature disordered skyrmion phase just below *B* = *B*_c_ (the magnetic-field value required to obtain the saturated uniform ferromagnetic state) reported in the bulk sample^20,21^ is absent in the present thin-plate sample.

Next, we investigate the magnetic-field variation of the quenched metastable SkL state. By performing a field cooling (FC) passing through the equilibrium SkL phase (black line in Fig. 2a: Path 1), the SkL phase can survive down to lower temperatures as a metastable state. Here, the nucleation probability generally scales with the sample volume and thus the first-order transition from the SkL phase to the competing conical phase accompanied by the change of topological number can be effectively avoided even with the relatively slow cooling ratio (10 K/min) in such a micro-fabricated crystal^22^. In Fig. 2e, the RSXS diffraction pattern obtained at 20 K just after the FC process at +60 mT (Path 1) is indicated. In the present setup, a magnetic field is applied along the out-of-plane [001] direction and the magnetic modulation vectors within the (001) plane is detected. The observed six-spot diffraction pattern (Fig. 2e) indicates the existence of three **Q**-vectors within a plane perpendicular to **B**, demonstrating the realization of the metastable triangular SkL. In the *B-*increasing process (Fig. 2d), the metastable triangular SkL state with the six-spot diffraction pattern remains up to 150 mT, i.e., just before entering the conical phase, where the **Q***-*vector is aligned parallel to the out-of-plane *B-*direction and diffraction spots disappear. In the *B-*decreasing process, the triangular SkL state survives even down to negative fields, whereas the six-spot pattern suddenly transforms into a four-spot pattern at −40 mT (Fig. 2f) and then the transition into the conical state takes place at −45 mT (Fig. 2g). The *B*–*T* phase diagram for the abovementioned field-sweeping process (Path 1) is summarized in Fig. 2a. In another field-sweeping process (black line in Fig. 2b: Path 2), where the magnetic field is returned back to the positive direction after the four-spot pattern once appeared at −40 mT, this four-spot pattern survives up to positive fields (Fig. 2j) and then the six-spot pattern is retrieved above 50 mT (Fig. 2i). From such a reversible change between the four-spot and six-spot patterns, the observed four-spot pattern is assigned to a square SkL state, endowed with the topological charge. Here, a multi-domain state of helical magnetic phase (i.e., coexistence of helical domains with **Q** || [100] and **Q** || [010]) with zero topological charge can be ruled out as the origin of the four-spot diffraction pattern, as the metastable triangular SkL state cannot be generated by sweeping the field from the equilibrium helical spin state at this temperature. For comparison, the field variation of the diffraction patterns at 20 K after ZFC is shown in Fig. 2k–m, where the helical state with two-spot pattern (**Q** || [100]) at zero field directly changes into the conical state (**Q** *||* **B**) in the field-increasing process and no trace of SkL state (${\mathbf{Q}} \bot {\mathbf{B}}$) is observed (Fig. 2c). (The results for the different field-sweeping path and the detailed analysis of the RSXS data are provided in Supplementary Notes II and III).

To investigate the microscopic origin of lattice transformation of SkL, we have performed micromagnetic simulations based on Landau–Lifshitz–Gilbert (LLG) equation by using MuMax3 software^23^. The stable magnetization distribution **m**(**r**) was deduced by minimizing the magnetic free energy $E = {\int} {\varepsilon \;d{\mathbf{r}}}$ with energy density *ε*(**r**) given by

2 $$\varepsilon = J[(\partial _x{\mathbf{m}})^2 + (\partial _y{\mathbf{m}})^2 + (\partial _z{\mathbf{m}})^2] + D{\mathbf{m}} \cdot (\nabla \times {\mathbf{m}}) + M_S{\mathbf{B}}_{{\mathrm{ext}}} \cdot {\mathbf{m}} - \frac{1}{2}M_S{\mathbf{B}}_{\mathrm{d}} \cdot {\mathbf{m}},$$

where the first, second, third, and fourth terms represent Heisenberg exchange, DM, Zeeman, and magnetostatic energy, respectively. *J*, *D*, and *M*_s_ represent the magnitudes of exchange interaction, DM interaction, and local magnetic moment, respectively. **B**_ext_ and **B**_d_ are the external magnetic field and the demagnetizing field, respectively. A small amount of impurity sites are randomly introduced in the present model (see “Methods” for the detail).

**Fig. 3:**
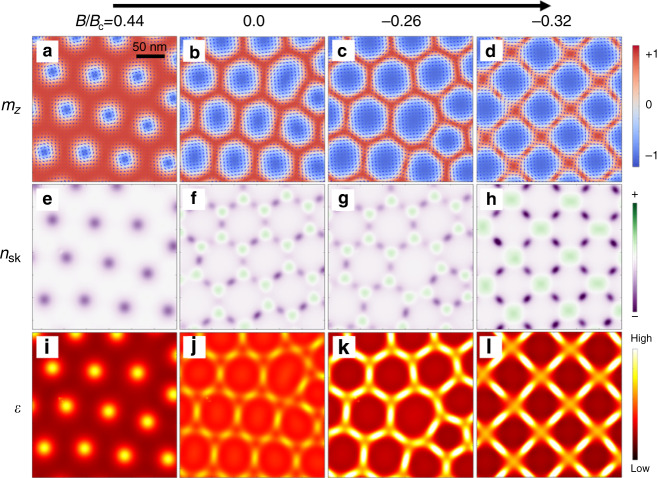
Magnetic-field-induced structural transition of metastable skyrmion lattice calculated by micromagnetic simulations. **a**–**d** The distribution of local magnetization **m** in the field-decreasing process from the triangular SkL state in **a**. The arrows correspond to the in-plane component of local magnetization and the background color represents the out-of-plane component of local magnetization (*m*_z_). **e**–**l** Contour map of (**e**–**h**) topological charge density (*n*_sk_) and (**i**–**l**) the energy density (*ε*) corresponding to **a**–**d**.

Figure 3 summarizes the real-space distribution of local magnetic moment **m** (Fig. 3a–d), topological charge density *n*_sk_ (Fig. 3e–h), and energy density *ε* (Fig. 3i–l) calculated with various amplitudes of magnetic field based on the micromagnetic simulations. Here, the triangular SkL state is initially prepared at *B/B*_c_ = +0.44 (Fig. 3a) and then the magnetic field is altered at zero temperature (Fig. 3b–d). At *B/B*_c_ = +0.44 (Fig. 3a), neighboring skyrmion cores (i.e., regions with negative out-of-plane moment *m*_*z*_, whose diameter is defined as *d*) are well separated and negative sign of *n*_sk_ is concentrated at the center of the core regions (Fig. 3e). For such a positive value of *B*, the skyrmion core region has higher energy density (Fig. 3i), reflecting the energy cost due to the Zeeman term. With decreasing the magnetic field, the skyrmion core region gradually expands and the intervening region between neighboring skyrmions (i.e., the region with positive *m*_*z*_) squeezes reflecting the modification of the Zeeman energy gain, while the total number of skyrmion cores and their triangular-lattice form are still kept unchanged at zero field (Fig. 3b) and even in the negative *B* (Fig. 3c). By further decreasing *B*, the transition into the square SkL state happens around *B/B*_c_ = −0.3, in accord with the experimental observation (Fig. 3d). Importantly, in case of the negative sign of *B*, the energy cost becomes largest at the interface region between two neighboring skyrmion cores (Fig. 3j–l). It is mainly because this region is characterized by (1) the positive sign of *m*_*z*_ with Zeeman energy cost, and (2) the steep spatial change of local moment direction, which disturbs the ideal spin modulation pitch determined by the balance between *J* and *D* and hence causes a large exchange energy cost. In particular, the latter contribution becomes more significant as the skyrmion core diameter *d* becomes larger (i.e., the intervening region becomes narrower), which demands the reduction of the number of energy-costing skyrmion–skyrmion interface. As the individual skyrmion particle is surrounded by six (four) skyrmions in the triangular (square) SkL, the energy cost at such interface regions between nearest-neighbor skyrmions can be reduced by the transition from triangular SkL into square SkL. Therefore, we can understand that the observed triangular-to-square transformation of SkL is mainly caused by the *B-*dependent modification of skyrmion core diameter *d* (In the abovementioned process, spin vortices and anti-vortices emerge at the intervening region between original skyrmion cores (Fig. 3f–h), whereas their contribution to *N*_sk_ cancels out and the total topological charge remains unchanged. See Supplementary Note VII).

**Fig. 4:**
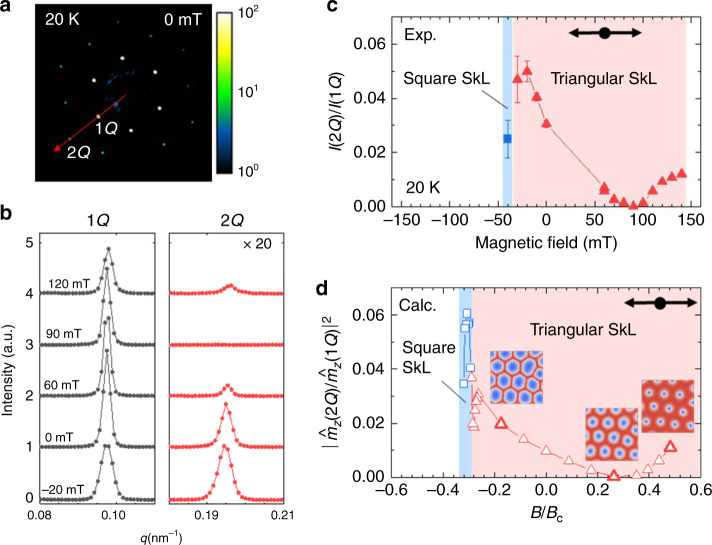
Magnetic-field dependence of anharmonicity in the SkL spin texture. **a** A RSXS diffraction pattern at 20 K for the metastable triangular SkL state at zero field after field cooling (Path 1). **b** The line-scan profile of scattering intensity for the fundamental (1*Q*) and the second-order harmonic (2*Q*) reflections along the red arrow in **a**. **c** Magnetic field dependence of the 2*Q* intensity (*I*(2*Q*)) normalized by the 1*Q* one (*I*(1*Q*)). The diffraction intensity is integrated so as to encompass the diffraction spots. Similar behavior has also been confirmed for other line-scan directions. **d** Magnetic field dependence of $|\hat m_z(2Q)/\hat m_z(1Q)|^2$ calculated from the results of micromagnetic simulations. The spin texture for the selected *B*-value (highlighted by bold triangular symbols) are also shown in the inset. Black arrows indicate the direction of field sweep.

In principle, such a deformation of skyrmion particle can be evaluated by measuring the relative amplitude of the higher harmonics in the magnetic modulation. Figure 4d indicates the magnetic-field dependence of $|\hat m_z(2Q)/\hat m_z(1Q)|^2$ calculated from the result of the micromagnetic simulation, with $\hat m_z(1Q)$ and $\hat m_z(2Q)$ representing the amplitude of fundamental (1*Q*) and second-order harmonic (2*Q*) modulation component of *m*_*z*_, respectively. As the *B-*value decreases in the triangular SkL state, the skyrmion core diameter *d* monotonically increases, whereas the core-to-core distance *a* remains constant to keep the total topological number unchanged. At *B/B*_c_ ~ +0.25, the second harmonic component $({\mathrm{i}}.{\mathrm{e}}.|\hat m_z(2Q)/\hat m_z(1Q)|^2)$ is minimized, where *d* is the half of *a* and the magnetic modulation is almost sinusoidal. When *B* becomes larger or smaller than this value, *d/a* deviates from 1/2 and the larger amplitude of second harmonics is induced. With decreasing *B*, the second harmonic component in the triangular SkL state reaches a maximum just before the transition into the square SkL state around *B/B*_c_ = −0.3. The square SkL is stable only for a narrow *B* range and the further decrease of *B* leads to the destruction of skyrmions.

To experimentally confirm such *B-*induced change of skyrmion core size, we investigate the second harmonic magnetic reflections in the RSXS results. It is noteworthy that the RSXS intensity mainly reflects the component of ${\hat{\mathbf{m}}}({\mathbf{Q}})$ parallel to the out-of-plane [001] direction (i.e., $\hat m_z({\mathbf{Q}})$), as discussed previously. In Fig. 4a, the diffraction pattern measured at 0 mT in the metastable triangular SkL state is indicated, where magnetic reflections corresponding to the 2*Q* modulation components are clearly discerned in addition to the 1*Q* ones. Figure 4b indicates the line-scan profile of diffraction intensities for the 1*Q* and 2*Q* reflections measured at 20 K with various amplitudes of *B*, and the magnetic-field dependence of *I*(2*Q*)/*I*(1*Q*), i.e., the integrated intensity of 2*Q* magnetic reflection normalized by the 1*Q* one, is summarized in Fig. 4c. *I*(2*Q*)/*I*(1*Q*) in the triangular SkL state exhibit the minimum at around +90 mT and a deviation of *B*-values from this minimum leads to the enhancement of second harmonic intensity. As *B* is further reduced, *I*(2*Q*)/*I*(1*Q*) ratio monotonously increases and reaches a maximum just before the transition into the square SkL phase at −40 mT. These experimental behaviors are in good agreement with the afore-mentioned prediction by the micromagnetic simulations (Fig. 4d), which suggests that the observed transition between the triangular and square SkL phases is indeed triggered by the *B-*induced modification of skyrmion core diameter (see Supplementary Note III for the detailed discussion on the *I*(2*Q*)/*I*(1*Q*) profile).

## Discussion

Previously, the stability of square SkL phase has been discussed in several theoretical works^2,20,24–26^, while they mostly focused on the case of the equilibrium ground state in the positive field region, i.e., not the non-equilibrium metastable state in the negative field region as studied here. As the simplest magnetic Hamiltonian in Eq. (2) generally favors the close-packed triangular SkL phase as the ground state, the additional contribution of magnetic anisotropy and/or higher-order four-spin interaction is required to stabilize the square SkL state in the equilibrium condition according to the previous reports^20,24–26^. On the other hand, our present calculation suggests that these interactions are not necessary to obtain the metastable square SkL state in the non-equilibrium condition, only if skyrmion particles can survive up to a sufficiently large amplitude of negative field during the *B-*sweeping process. The overall good agreement between the theoretical and experimental results (i.e., the non-equilibrium phase diagram and *B-*dependence of skyrmion core diameter as shown in Fig. 4c, d) supports the validity of this picture, demonstrating that the *B-*dependent modification of skyrmion core diameter and associated energy cost at the skyrmion–skyrmion interface region are the key for the observed triangular-to-square SkL transformation. It is noteworthy that for the present case with insulating Cu_2_OSeO_3_, the contribution of four-spin interaction (that is generally mediated by itinerant electrons) is negligible, whereas the magnetic anisotropy may also cooperatively promote the appearance of metastable square SkL on the (001) plane. The latter contribution affects the critical *B*-value for the triangular-to-square SkL transition (see Supplementray Note IV. The discussion on the temperature dependence is also provided in Supplementary Note VI).

In the present work, we have clarified the detailed mechanism of magnetic-field-induced structural change of metastable SkL. The observed phase transition process is unique, since it is accompanied by the significant *B*-induced size change of skyrmion particle, in contrast with the case of conventional atomic or molecular crystals. Interestingly, a similar field-induced size/shape change has also been reported for other topological solitons such as heliknotons in liquid crystal systems recently^10^, where the electric-field-induced lattice symmetry change and giant electrostriction are observed. Our present results suggest that topological solitons characterized by the flexible particle-shape/size degree of freedom commonly host unique manner of crystallization and field-induced structural phase transition, which may promise emergence of intriguing phenomena and functions from topological-particle ensembles.

## Methods

### Sample preparation

A single crystal of Cu_2_OSeO_3_ was grown by a chemical vapor transport method. A (001)-oriented thin plate of Cu_2_OSeO_3_ with about 800 nm thickness was prepared using the focused ion beam (FIB) microfabrication technique (Supplementary Fig. 6). To block the transmission beam, the back side of the Si_3_N_4_ membrane window was covered with gold film, and subsequently, a pinhole of about 6 μm in diameter was drilled. The sample was mounted to cover the pinhole and attached to the membrane with single tungsten contact to avoid tensile strain.

### Small-angle RSXS

Small-angle RSXS experiments were performed using circularly polarized X-ray with the resonance energy of 931 eV (i.e., Cu *L*_3_ absorption edge) in the transmission geometry at the beamline BL-16A, Photon Factory, KEK, Japan^27^. The diffraction patterns were recorded by a direct-detection CCD detector, which was protected from the transmitted direct beam with a beam catcher. The magnetic field was applied parallel to the incident X-ray beam and perpendicular to the thin plate (|| [001]) by a Helmholtz coil. We note that the orientation of the SkL is stochastic and sometimes accompanied by a multi-domain SkL state as previously reported in soft X-ray scattering experiments in the thin-plate samples^22^ and bulk samples^28,29^.

### Micromagnetic simulation

The micromagnetic simulations based on LLG equation were performed with varying bias field by using MuMax3 software^23^. The magnetization distribution for each value of the bias field was deduced by minimizing the magnetic free energy in Eq. (2). Here we used the material parameters, *J* = 8.78 × 10^−12^ Jm^−1^, *D* = 1.58 × 10^−3^ Jm^−2^, *M*_s_ = 3.84 × 10^5^ Am^−1^, and Gilbert damping constant *α* = 0.1, reported for FeGe^30^, which hosts similar magnetic modulation period as Cu_2_OSeO_3_. In this case, the magnetic-field value required to obtain the saturated uniform ferromagnetic state *B*_c_ = 1.14 T. The simulation program was run for a system with a size of 1024 × 1024 × 2 nm^3^ modeled with mesh sizes of 2 × 2 × 2 nm^3^ under periodic boundary conditions along the *x* and *y* axes. To represent impurities and/or defects due to Ga ion irradiation arising from FIB fabrication process in the present sample, we introduced the easy-plane magnetic anisotropy *K* = 1 × 10^6^ Jm^−3^ at randomly selected sites and set the density of the random impurities to 0.014%. Such defect sites are known to enhance the metastability of SkL state by preventing the first-order phase transition into the thermodynamically stable non-topological magnetic state^31^. The initial magnetization distribution at *B/B*_c_ = 0.44 (Fig. 3a) was prepared by relaxing a spin configuration of a triangular SkL constructed by using the built-in function of MuMax3. The simulation results for the thicker sample is provided in Supplementary Note V.

## Supplementary information

Supplementary Information

## Data Availability

The data presented in the current study are available from the corresponding authors on reasonable request.
