# Supplementary material for: Particle-size dependent structural transformation of skyrmion lattice
Source: Nat Commun. 2020 Nov 11;11:5685. doi: 10.1038/s41467-020-19480-8 (PMC7658213; doi:10.1038/s41467-020-19480-8)
Supplement: Supplementary file 1 — Supplementary Information [file 41467_2020_19480_MOESM1_ESM.pdf]

Supplementary Information for

Particle-size dependent structural transformation of skyrmion lattice

Authors

R. Takagi[1,2,3*], Y. Yamasaki[1,4,5,6], T. Yokouchi[1], V. Ukleev[1,7], Y. Yokoyama[8], H. Nakao[6], T. Arima[1,9], Y. Tokura[1,2,10], S. Seki[1,2,3,5]

Affiliations

[1] RIKEN Center for Emergent Matter Science (CEMS), Wako 351-0198, Japan

[2] Department of Applied Physics, University of Tokyo, Tokyo 113-8656, Japan,

[3] Institute of Engineering Innovation, University of Tokyo, Tokyo 113-0032, Japan,

[4] Research and Services Division of Materials Data and Integrated System (MaDIS), National Institute for Materials Science (NIMS), Tsukuba, 305-0047 Japan,

[5] PRESTO, Japan Science and Technology Agency (JST), Kawaguchi 332-0012, Japan,

[6] Condensed Matter Research Center and Photon Factory, Institute of Materials Structure Science, High Energy Accelerator Research Organization, Tsukuba 305-0801, Japan,

[7]Laboratory for Neutron Scattering and Imaging (LNS), Paul Scherrer Institute (PSI), CH-5232 Villigen, Switzerland,

[8]Japan Synchrotron Radiation Research Institute (JASRI/SPring-8), Sayo 679-5198, Japan,

[9] Department of Advanced Materials Science, University of Tokyo, Kashiwa 277-8561, Japan,

[10]Tokyo College, University of Tokyo, Tokyo 113-8656, Japan.

*corresponding author. E-mail: takagi@ap.t.u-tokyo.ac.jp

Supplementary Note I: Identification of equilibrium SkL state

To confirm the magnetic field (B)-temperature (T) phase diagram in the equilibrium condition, we performed the small-angle resonant soft x-ray scattering (RSXS) measurements in the field sweeping process after a zero-field cooling (ZFC) (Fig. 2c in the main text). As reported in the previous works, a helical phase (Fig. 1e in the main text) is realized for $B = 0$, where the neighboring spins are rotating within a plane normal to the magnetic modulation vector **Q** || <100>. Since the present RSXS measurement detects the Q-vector within the (001) plane, a pair of diffraction spots along the [100] direction appears at zero field (Fig. 2m in the main text). With increasing **B** || [001], the conical state (Fig. 1d in the main text) is stabilized, in which the magnetic modulation vector is aligned parallel to the magnetic field (i.e. **Q** || **B**) and no diffraction spot appears in the scattering plane (Fig. 2k in the main text). For limited temperature range just below T_c, a triangular SkL state with a hallmark six-spot diffraction pattern (i.e. $\mathbf{Q} \perp \mathbf{B}$) is also observed around 30 mT as shown in Supplementary Fig. 1c. Similar field-sweeping measurements were performed at various temperatures, and the phase boundaries of the equilibrium SkL state were determined as green regions in Fig. 2c in the main text.

Supplementary Note II: Phase boundary of metastable SkL state

In the field sweeping process indicated by the arrow in Supplementary Fig. 1a (Path 2), where the magnetic field was returned back to the positive direction once the four-spot pattern appeared at -40 mT, the four-spot pattern (square SkL) survived up to the positive fields (Supplementary Fig. 1h) and then the six-spot pattern (triangular SkL) was retrieved above 50 mT (Supplementary Fig. 1g). On the other hand, when the magnetic field was returned back to the positive direction from -45 mT (Supplementary Fig. 1b, Path 3), just after the four-spot pattern disappeared (Supplementary Fig. 1f), neither six-spot nor four-spot pattern was retrieved (Supplementary Fig. 1i). Thus, the phase boundary of metastable SkL state can be depicted as the dashed line in Supplementary Fig. 1b.

Supplementary Note III: Detailed comparison between RSXS data and simulation results

To analyze the field dependence of metastable SkL state in detail, the area-integrated intensity of RSXS magnetic diffraction spots for the field-sweeping process

of Path 1 and Path 2 are displayed in Supplementary Figs. 2a and c, respectively. For Path 1, on increasing the magnetic field, the six-spot intensity due to the triangular SkL suddenly turns into zero at around +160 mT, indicating that the triangular SkL directly changes into the conical state. On decreasing the magnetic field, the triangular SkL state with six-spot diffraction pattern was replaced by the square SkL state with four-spot pattern at around -40 mT, accompanied by a reduction of the total intensity. In the returning process (Path 2), the total intensity is further reduced through the transition from the square SkL state to the triangular SkL state at +45 mT. Such reduction of the intensity implies that some part of the metastable skyrmion domains were collapsed and transformed into the conical state when crossing the transformation of SkL.

To understand such behaviors at the phase boundary, in the following, we discuss the results of our micromagnetic simulation (as introduced in the main text) in detail. Supplementary Figures 3a-c indicate the theoretically calculated magnetic field dependence of total skyrmion number N_{sk}, the amplitude of fundamental peak in fast Fourier transform (FFT) image $|\hat{m}_z(1Q)|^2$ (corresponding to $I(1Q)$ in RSXS experiments), and wavenumber q for Path 1. At the triangular-to-square transition of metastable SkL, the skyrmion number is kept unchanged and the q value slightly decreases. By further decreasing the magnetic field value, partial destruction of skyrmions leads to gradual suppression of N_{sk} and $|\hat{m}_z(1Q)|^2$ with further reduction of q. Even in the latter situation, the overall square ordering of SkL and the associated four-fold scattering pattern are still sustained (Supplementary Figs. 3e and g), until the first-order transition into the non-topological magnetic state is completed.

Experimentally, the observed triangular-to-square SkL transition is accompanied with the considerable suppression of $I(1Q)$ and small decrease of q-value (Supplementary Figs. 2a and b). This behavior is consistent with the one expected for the aforementioned square SkL state at the phase boundary, where the slightly disordered square SkL state as shown in Supplementary Figs. 3e and g is probably realized at -40 mT (Fig. 2f in the main text). Note that we also tried to detect the predicted perfect square SkL in the experiments, while it turned out to be very difficult due to the narrow B-range of square SkL phase. Nevertheless, the overall good agreement between the theory and experiment suggests that our model well captures the physics behind the present system.

In Fig. 4d in the main text, the theoretically expected B-dependence of relative amplitude of the second harmonic intensity for Path 1 is plotted. The relative amplitude of the second harmonic intensity is increased just after the triangular-to-square transformation of SkL, but then decreased again by a subtle additional sweep of

magnetic field, reflecting the partial destruction of metastable skyrmions and associated change of skyrmion density. The observed suppression of $I(2Q)/I(1Q)$ value at the triangular-to-square transformation of SkL in the RSXS experiments (Fig. 4c in the main text) probably detects such deformation process of SkL.

For Path 2, we found that the q value in the triangular SkL state is considerably suppressed as the magnetic field increases in the RSXS experiments (Supplementary Fig. 2d). When skyrmion clusters coexist with the conical state, the core-to-core distance of neighboring skyrmions is gradually enhanced as a function of B according to Supplementary Ref. [1]. The behavior of q for Path 2 probably reflects such a process.

Supplementary Note IV: Influence of magneto-crystalline anisotropy

In this part, we discuss the effect of magnetic anisotropy on the magnetic-field-induced transformation of metastable SkL, as hinted by the previous reports that the amplitude of cubic magneto-crystalline anisotropy in Cu_2OSeO_3 is enhanced at low temperatures [2]. For this purpose, we further consider the effect of cubic magneto-crystalline anisotropy on the original model of Eq. (2) in the main text. The energy density associated with the cubic anisotropy $\varepsilon_{\mathrm{cubic}}$ is given by [3]

$$\varepsilon_{\mathrm{cubic}} = K_c[(\mathbf{c}_1 \cdot \mathbf{m})^2(\mathbf{c}_2 \cdot \mathbf{m})^2 + (\mathbf{c}_1 \cdot \mathbf{m})^2(\mathbf{c}_3 \cdot \mathbf{m})^2 + (\mathbf{c}_2 \cdot \mathbf{m})^2(\mathbf{c}_3 \cdot \mathbf{m})^2] \quad \text{(S1)}$$

where K_c is the first order cubic anisotropy constant and $\boldsymbol{c}_1$, $\boldsymbol{c}_2$, and $\boldsymbol{c}_3$ is a set of mutually perpendicular unit vectors indicating the anisotropy directions (cubic axes). Here, we used K_c = -600 J/m^3, which corresponds to the maximum amplitude of K_c experimentally identified for Cu_2OSeO_3 in the preceding studies [2,4].

Supplementary Figures 4a-c show the real-space distribution of local magnetic moment **m** calculated for various B values based on the micromagnetic simulations. The system is initially in the triangular SkL state (Supplementary Fig. 4a) and the neighboring skyrmion cores are well separated. On decreasing the B value, the skyrmion core diameter gradually expands with keeping the triangular lattice form down to the negative B (Supplementary Fig. 4b). By further decreasing B, the transition into the square SkL state was found (Supplementary Fig. 4c). These results are summarized in the phase diagram in Supplementary Fig. 4d, which turned out to be almost the same as the simulated results in the main text that do not consider the $\varepsilon_{\mathrm{cubic}}$ term. This supports that the observed triangular-to-square transformation of metastable SkL is mainly triggered by the B-induced modification of skyrmion core diameter. It also suggests that magnetic anisotropy is not always necessary for the formation of square SkL, if the skyrmions can survive up to a sufficiently large magnitude of negative field. Note that

the critical B-value for the triangular-to-square SkL transition is slightly shifted to the positive direction, suggesting a cooperative contribution of the cubic magneto-crystalline anisotropy to the stabilization of metastable square SkL state.

Supplementary Note V: Simulations for the three-dimensional system

To confirm the reproducibility of the triangular-to-square SkL transition in the three-dimensional system, we have performed a micromagnetic simulation for a system with a thickness of 8 cells, corresponding to 16 nm. Here, in our model of Eq. (2) in the main text, only magnetostatic term depends on the sample thickness, and the other terms, that is, the exchange, DM, and Zeeman terms, are of bulk origin. Using the same material parameters and impurity density as described in the Method section of the main text, we have reproduced the magnetic-field-induced deformation of skyrmion core, followed by the triangular-to-square lattice transformation of metastable skyrmions, as summarized in Supplementary Fig. 5. The same tendency as the result of the thinner system supports the validity of our scenario that B-dependent modification of skyrmion core diameter plays the key role in the observed triangular-to-square SkL transformation.

Supplementary Note VI: Temperature dependence

For the present Cu_2OSeO_3, the necessary condition for the appearance of square SkL is the survival of metastable skyrmions down to the sufficiently large amplitude of negative magnetic field, where the B-dependent expansion of skyrmion core diameter and associated energy cost at the skyrmion-skyrmion interface induce the triangular-to-square SkL transition. At higher temperature, the additional thermal fluctuation promotes the transition from the metastable SkL state to thermodynamically stable non-topological magnetic state, and therefore skyrmions collapse before reaching the critical magnetic field value ($B_{t\text{-}s}$) necessary for the triangular-to-square SkL transition. Note that this critical $B_{t\text{-}s}$ value also depends on temperature, because of the change of the balance between the amplitude of thermal fluctuation and energy barrier between the triangular and square SkL states. Such behaviors, including the disappearance of metastable square SkL state at higher temperature, are indeed observed in the non-equilibrium B-T phase diagram of Cu_2OSeO_3 for Path 1 (Fig. 2a in the main text). Similar temperature dependent behavior has also been observed in Supplementary Ref. [5].

Supplementary Note VII: Appearance of additional vortices and anti-vortices with fractional topological charges

As seen in Figs. 3f-h in the main text, as the skyrmion core region expands with decreasing the magnetic field, there appear additional vortices and anti-vortices carrying topological charge density of positive and negative signs at the intervening region between original skyrmion cores, respectively. Here, the topological charges of these vortices and anti-vortices are fractional, and their contributions to the skyrmion number cancel out with keeping the total topological charge unchanged. Considering these vortices and anti-vortices as fractionalized topological "particles", the spin configurations in Figs. 3f-h may be viewed as the precursor of the lattice of merons /anti-merons with $N_{\mathrm{sk}} = \pm 1/2$, as proposed in Supplementary Ref. [6]. The further interpretation based on such a picture, as well as the elucidation of interactions between these fractionalized topological objects, is an issue for the future study.

Supplementary Note VIII: Sample Preparation

For the small-angle resonant soft X-ray scattering (RSXS) experiments, a plate-shaped sample was extracted from the bulk single crystal and carefully thinned to make the flat surface by using focused ion beam (FIB) microfabrication technique. The size of the thin plate is 18 μm × 17 μm × 800 nm. A scanning electron microscope (SEM) image of the thin-plate sample is shown in Supplementary Fig. 6.

Reference

[1] Yu, X. Z. et al. Aggregation and collapse dynamics of skyrmions in a non-equilibrium state. *Nat. Phys.* **14**, 832 (2018).

[2] Halder, M., et al., Thermodynamic evidence of a second skyrmion lattice phase and tilted conical phase in Cu_2OSeO_3. *Phys. Rev. B* **98**, 144429 (2018).

[3] Vansteenkiste, A., Leliaert, J., Dvornik, M., Helsen, M., Garcia-Sanchez, F. & Van Waeyenberge, B. The design and verification of MuMax3. *AIP Adv.* 4, 107133 (2014).

[4] Stasinopoulos, I., et al., Low spin wave damping in the insulating chiral magnet Cu_2OSeO_3. *Appl. Phys. Lett.* **111**, 032408 (2017).

[5] Nakajima, T. et al. Skyrmion lattice structural transition in MnSi. *Sci. Adv.* **3**, e1602562 (2017).

[6] Lin, S-Z., Saxena, A. & Batista, C. D. Skyrmion fractionalization and merons in chiral magnets with easy-plane anisotropy. *Phys. Rev. B* **91**, 224407 (2015).

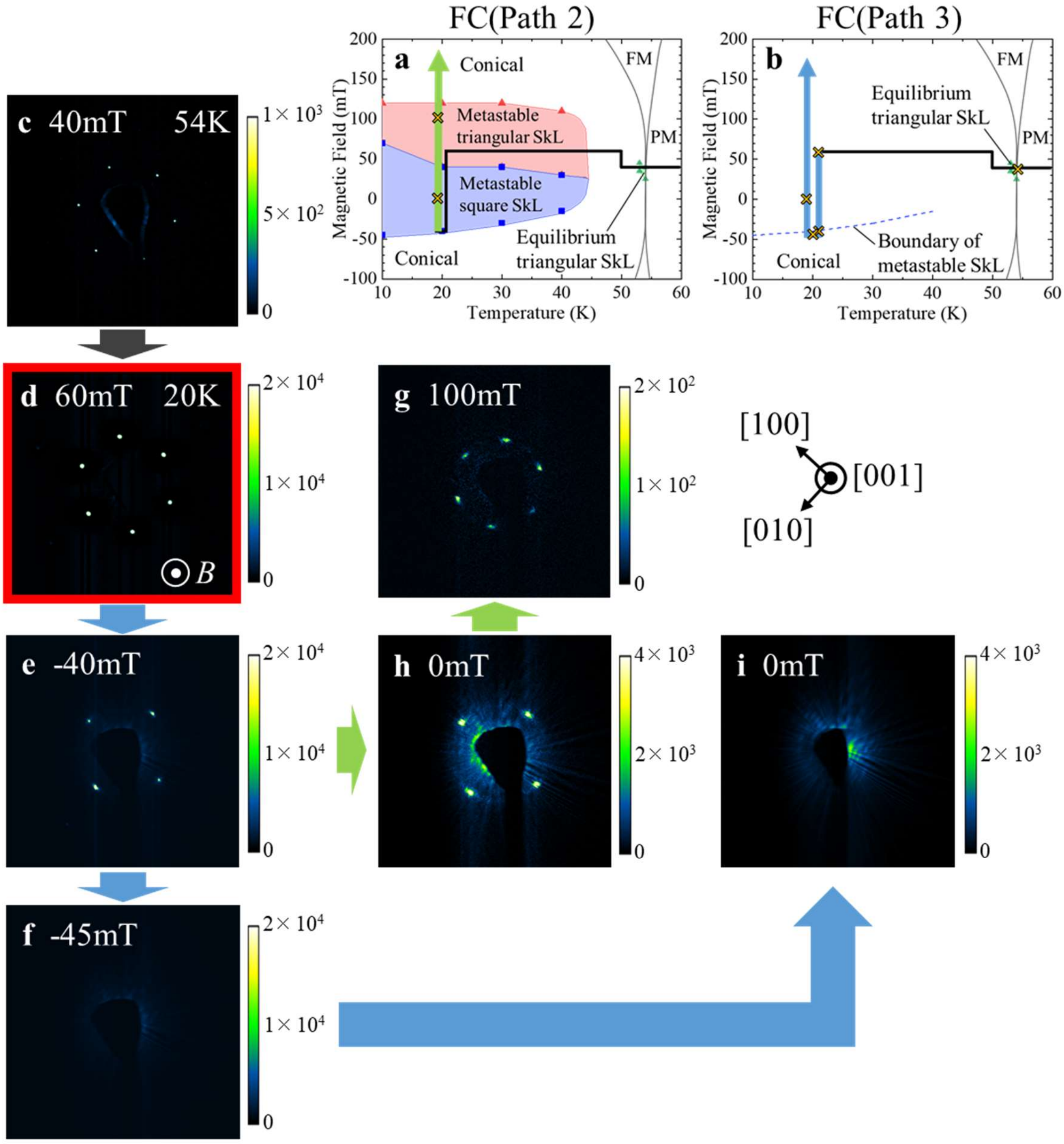

Supplementary Figure 1 │ Magnetic field dependence of the RSXS diffraction patterns for a (001)-oriented Cu_2OSeO_3 thin plate. a,**b**, Magnetic field (*B*)-temperature(*T*) phase diagrams for **B** || [001] determined from the different manners of field-sweeping runs (Path 2 and Path 3, respectively) after field cooling. FM and PM represent the ferromagnetic and paramagnetic states, respectively. **c**, A diffraction pattern in the equilibrium triangular SkL state. **d-i**, Diffraction patterns taken at 20 K with various amplitudes of magnetic field for **B** || [001] in the field-sweeping process of Path 2 (**d-e**) and Path 3 (**f-i**). The color scales indicate the scattering intensity. Arrows between these figures represent the direction of field sweep, which correspond to the arrows in **a** and **b**.

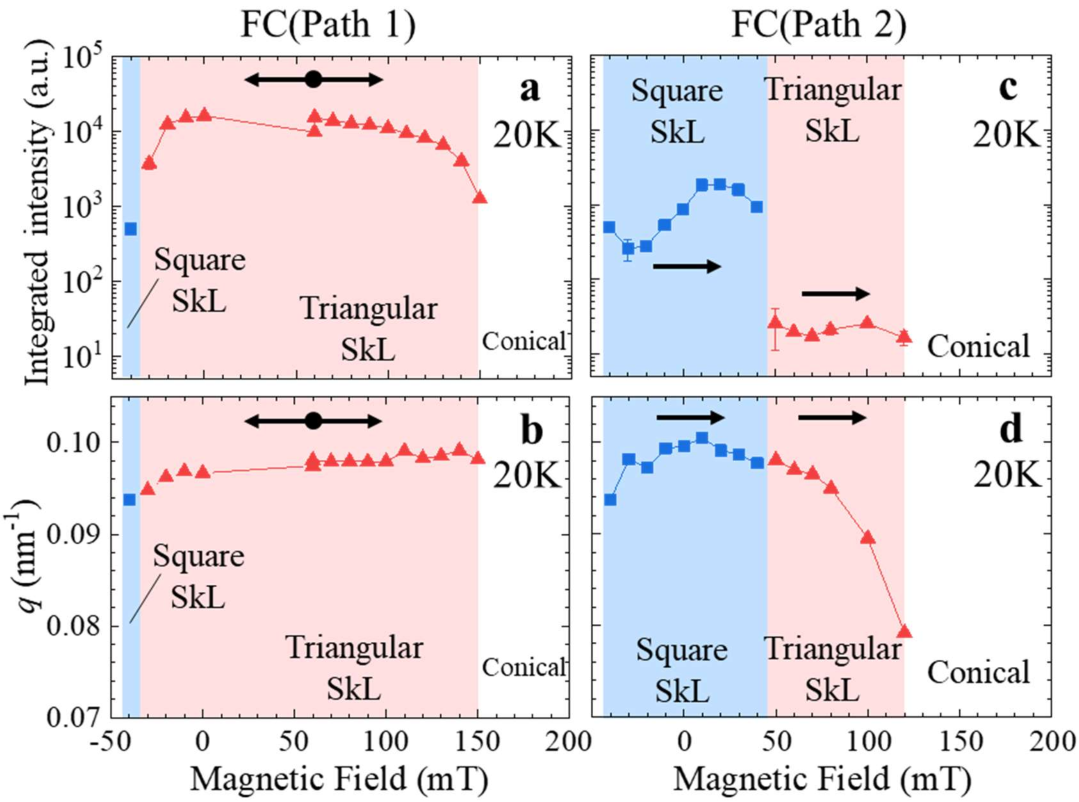

Supplementary Figure 2 │Magnetic field dependence of the metastable SkL. The integrated intensity and the wave number q of RSXS magnetic reflections as a function of magnetic field **B** || [001], measured for the Path 1 (Fig. 2a in the main text) (**a**,**b**) and Path 2 (Fig. 2b in the main text) (**c**,**d**). Triangular and square symbols represent the data in the metastable triangular and square SkL states, respectively. Field sweeping directions are denoted by black arrows.

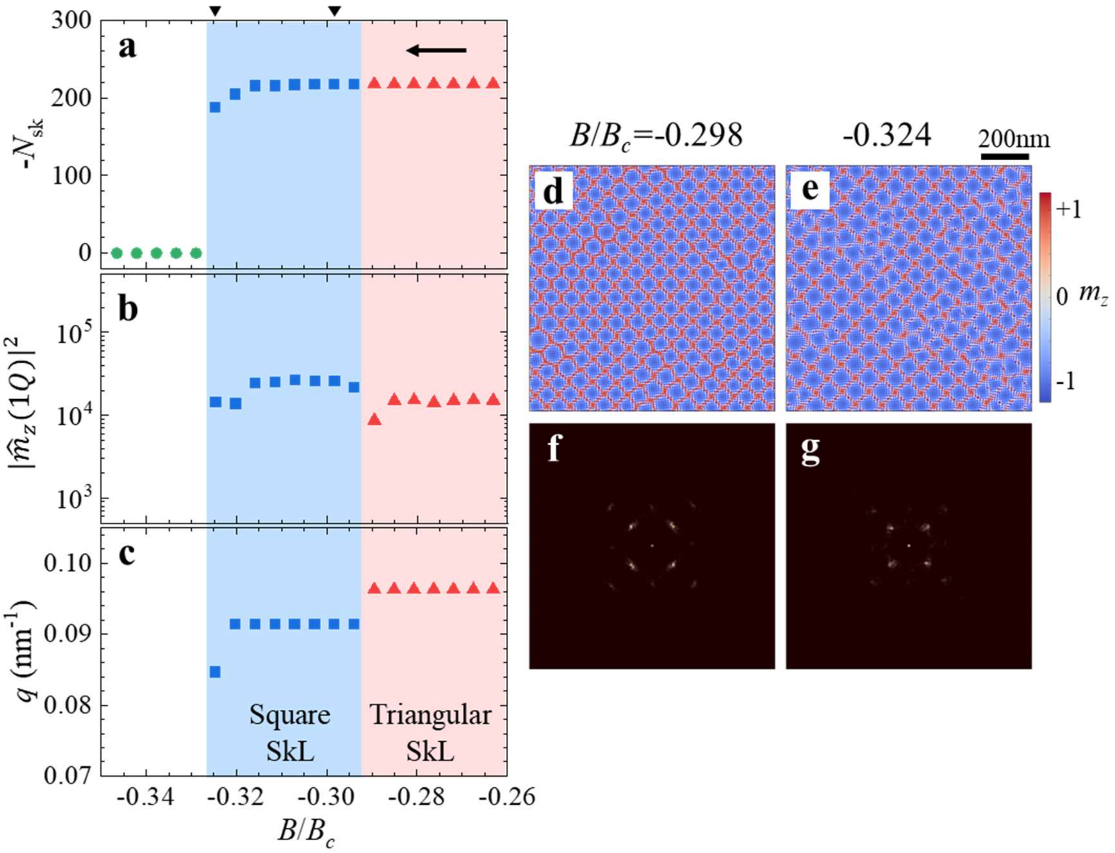

Supplementary Figure 3 │Magnetic field dependence of the metastable SkL around the structural transition of SkL. a-c, Magnetic field dependence of (**a**) skyrmion number (N_{sk}), (**b**) amplitude of fundamental peak in fast Fourier transform (FFT) image $|\hat{m}_z(1Q)|^2$, and (**c**) wave number (q) calculated from the results of micromagnetic simulation based on Eq. (2) in the main text for Path 1. Triangular and square symbols represent the data in the triangular and square SkL states, respectively. Field sweeping directions are denoted by black arrows. In **b**, the decrease of $|\hat{m}_z(1Q)|^2$ near the transition from triangular to square SkL state is due to appearance of square SkL. **d**,**e**, Real-space spin configuration for the selected B-value (indicated by triangular symbols at the upper side of **a**). The color represents the out-of-plane component of local magnetization (m_z). **f**,**g**, The FFT images corresponding to (**d**,**e**).

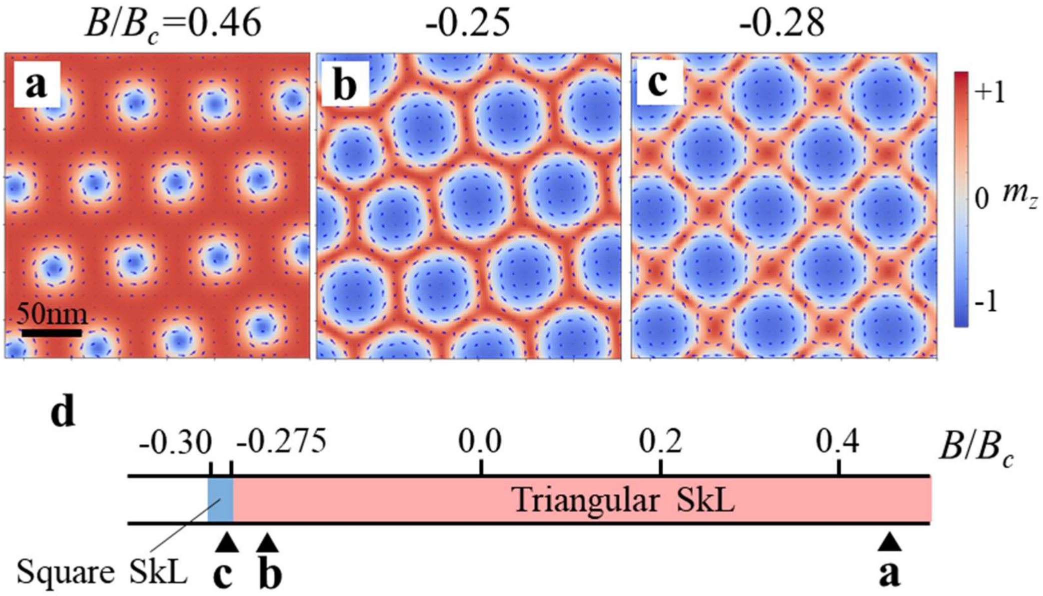

Supplementary Figure 4 │Micromagnetic simulation with magnetic anisotropy. a-c, The real-space distribution of local magnetization **m** obtained by micromagnetic simulation based on Eq. (2) in the main text with additional cubic magneto-crystalline anisotropy term K_{c1} = -600 J/m^3 (Eq. (S1)) [2,4] for various amplitudes of magnetic field applied through Path 1. The arrows (background colors) represent the in-plane (out-of-plane) component of local magnetization. **d**, Phase diagram for the above simulation as a function of B/B_c. The field values corresponding to (**a**-**c**) are marked by triangular symbols in **d**.

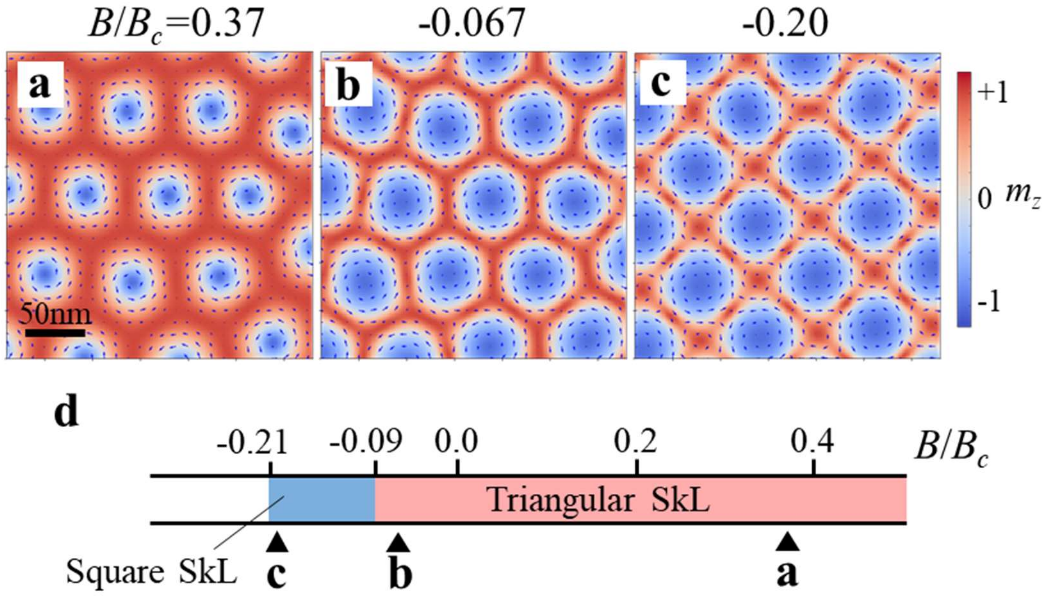

Supplementary Figure 5 | Micromagnetic simulation for the three-dimensional system. a-c, The distribution of local magnetization **m** calculated for the three-dimensional system with the thickness of 8 cells, based on the Eq. (2) in the main text with various amplitudes of magnetic field applied through Path 1. **d**, Phase diagram for the above simulations as a function of B/B_c. The field values corresponding to (**a**-**c**) are marked by triangular symbols in **d**.

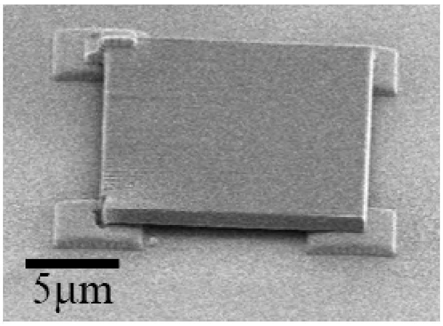

Supplementary Figure 6 | Sample for the small-angle RSXS experiments. The sample was prepared using the FIB microfabrication technique.
